# Myocardial fibrosis is a predictor of atrial fibrillation in dilated cardiomyopathy. Role of cardiovascular magnetic resonance

**DOI:** 10.1186/1532-429X-13-S1-P298

**Published:** 2011-02-02

**Authors:** Ana G Almeida, Joao S Marques, Maria-Jose Correia, Dulce A Brito, Claudio David, Doroteia R Silva, Claudia Jorge, Antonio N Diogo

**Affiliations:** 1 University Hospital Santa Maria, Lisbon, Portugal

## Introduction

Late gadolinium enhancement (LGE) by cardiovascular magnetic resonance (CMR) has been found in about two-thirds of patients with dilated cardiomyopathy (DCM). The clinical impact of this finding is not well established.

## Purpose

The aim of this study was to assess, in patients (pts) with DCM, the relationship of LGE with the occurrence of atrial fibrillation (AF), which is a common complication of DCM.

## Methods

44 consecutive pts (38±11 years-old, 29 men) with DCM were included, after exclusion of ischemic heart disease (coronary angiography), secondary cardiomyopathies (clinical and laboratory investigation), non-sinus rhythm and contraindications to CMR. Functional class and plasmatic NT-pro-BNP were assessed. All pts underwent CMR: a) short-axis SSFP for left atrial area and left ventricle (LV) volumes and ejection fraction (EF); b) LGE (segmented inversion-recovery fast gradient-echo sequence), 10-15 mn after 0.2mmol/kg of Gd-DTPA acquired in long-axis and short-axis views; LGE was classified as subendocardial or midwall and quantified using plannimetry of LGE contours, 6SD from remote myocardium; a fibrosis index was obtained from LGE volume/myocardial volume. All underwent 24-hours Holter monitoring, less than one month from CMR, and the number of atrial fibrillation episodes was assessed.

## Results

26 pts were in NYHA class II and 18 in class III. Mean NT-pro-BNP was 551±180pg/ml, atrial area was 35.8±11.0 cm2, LV end-diastolic volume was 155±44mL/m2, mean EF was 33±8%. LGE was found in 29 patients (69%) located in midwall, involving a mean of 7 segments per pt (range 3-14). Holter monitoring showed AF episodes in 26 pts. In comparison with pts without AF, pts with AF had higher NT-pro-BNP (836±110 vs 474±132 pg/ml, p=0.01), larger atrial area (44.1±10.1 vs 31.2±9,2 cm2, p=0.002), larger LV end-diastolic volumes (172±31 vs 153±35ml/m2, p=0.03) and more frequent LGE (p=0.0003). In pts with AF, no correlation was found between the functional class or the fibrosis index and the number of AF episodes. Using multivariate analysis, the atrial area and the presence of LGE were independent predictors of occurrence of AF episodes.

## Conclusion

In patients with DCM, LGE together with atrial dimensions, is significantly associated and an independent predictor of atrial fibrillation, among functional class, LV volumes, ejection fraction and NT-pro-BNP. This finding should be used for risk assessment and therapeutic decisions.

